# A Novel One-Pot Enzyme Cascade for the Biosynthesis of Cladribine Triphosphate

**DOI:** 10.3390/biom11030346

**Published:** 2021-02-25

**Authors:** Julia Frisch, Tin Maršić, Christoph Loderer

**Affiliations:** 1 Chair for Molecular Biotechnology, Technical University, 01217 Dresden, Germany; julia.frisch@mailbox.tu-dresden.de; 2 Laboratory for Genome Engineering and Synthetic Biology, Division of Biological Sciences, 4700 King Abdullah University of Science and Technology, Thuwal 23955-6900, Saudi Arabia; tin.marsic@kaust.edu.sa

**Keywords:** enzymatic nucleotide synthesis, enzymatic cascade synthesis, deoxyribonucleoside-5′-triphosphate, nucleotide analogue, adenine phosphoribosyltransferase, polyphosphate kinase, ribonucleotide reductase

## Abstract

Cladribine triphosphate is the active compound of the anti-cancer and multiple sclerosis drug Mavenclad (cladribine). Biosynthesis of such non-natural deoxyribonucleotides is challenging but important in order to study the pharmaceutical modes of action. In this study, we developed a novel one-pot enzyme cascade for the biosynthesis of cladribine triphosphate, starting with the nucleobase 2Cl-adenine and the generic co-substrate phosphoribosyl pyrophosphate. The cascade is comprised of the three enzymes, namely, adenine phosphoribosyltransferase (APT), polyphosphate kinase (PPK), and ribonucleotide reductase (RNR). APT catalyzes the binding of the nucleobase to the ribose moiety, followed by two consecutive phosphorylation reactions by PPK. The formed nucleoside triphosphate is reduced to the final product 2Cl-deoxyadenonsine triphosphate (cladribine triphosphate) by the RNR. The cascade is feasible, showing comparative product concentrations and yields to existing enzyme cascades for nucleotide biosynthesis. While this study is limited to the biosynthesis of cladribine triphosphate, the design of the cascade offers the potential to extend its application to other important deoxyribonucleotides.

## 1. Introduction

On 29 March 2019 the pharmaceutical Mavenclad (cladribine) was approved by the U.S. Food and Drug Administration (FDA) as treatment against relapsing forms of multiple sclerosis. Initially, cladribine was developed in the 1980s as a treatment for different forms of leukemia [1], and only 30 years later, its potential against multiple sclerosis was realized and studied in detail [2,3]. As a deoxyadenosine analogue, its mode of action revolves around its cytotoxicity by induction of DNA strand breaks [3,4]. Despite its long history, the detailed modes of action are subject of current research [4,5].

Cladribine or 2Cl-deoxyadenosine is a prodrug, whose pharmaceutically active metabolite is cladribine triphosphate [4]. In order to study the specific effect of cladribine triphosphate and similar deoxyribonucleotide compounds, an efficient synthesis procedure is required. The synthesis of non-natural deoxyribonucleotides, however, is difficult and expensive, illustrated by the respective prices (78,220 €/g Jena Bioscience, 08.01.2021). Novel strategies for the synthesis of such compounds are required to improve their availability and help us to understand their mode of action.

For the synthesis of non-natural nucleotides, several biocatalytic strategies have been developed [6]. In general, the process can be separated into two steps. First, the desired nucleobase is loaded on the ribose, or deoxyribose moiety. Then, the resulting nucleoside or nucleotide is phosphorylated to the desired phosphorylation level.

For the first step, several different types of enzymes are available. Purine nucleoside phosphorylases catalyze the transfer of a nucleobase on (deoxy)ribose-1-phopshate resulting in the formation of a nucleoside and phosphate release [7,8]. 2′-Deoxyribosyl transferases catalyze the exchange of the nucleobase moiety on a deoxyribonucleotide with a free nucleobase [9,10,11]. Both reactions can produce non-natural (deoxy)ribonucleosides such as 2Cl-adenosine [7,11]. Phosphoribosyltransferases catalyze the reaction of phosphoribosyl pyrophosphate (PRPP) with a nucleobase, yielding a nucleoside monophosphate and pyrophosphate [12,13,14,15,16,17].

Starting with the nucleoside or the nucleoside-monophosphate, different kinases have been applied for phosphorylation in order to synthesize the desired ribonucleoside triphosphate. Examples are adenylate kinases, creatine kinases, or pyruvate kinases [18,19,20]. With a combination of different kinases, the phosphorylation of nucleosides to the corresponding mono-, di-, and triphosphate derivatives was achieved, including the two natural deoxyribonucleotides dCTP and dATP [21]. Although existing methods are not strictly limited to ribonucleotides, an efficient biosynthesis of the non-natural deoxyribonucleotide cladribine triphosphate has not been reported so far. New strategies, specifically designed for the biosynthesis of non-natural deoxyribonucleotides, will be required to fill this gap.

Polyphosphate kinases (PPK) have been developed as a tool for ATP-regeneration [22,23,24,25,26,27]. They catalyze the transfer of phosphate groups from polyphosphate to nucleotides with different phosphorylation level [28]. Particularly interesting are PPK2 class III enzymes, capable of the double phosphorylation of AMP to ATP [29,30]. Given the necessary substrate promiscuity, this enzyme could single-handedly catalyze the phosphorylation of a non-natural nucleoside-monophosphate to the corresponding nucleoside triphosphate.

Another class of enzymes that are interesting in this regard are ribonucleotide reductases. They catalyze the reduction of nucleotides to the corresponding deoxyribonucleotides and are responsible for the de novo biosynthesis of the DNA building blocks in nature [31]. Depending on the class of RNR, they catalyze the reduction of either nucleoside di- or triphosphates [32,33,34]. In principle, they are capable of reducing different natural nucleotides but the substrate specificity is subject to allosteric regulation. Binding of a specific dNTP at the allosteric site activates the enzymes for the conversion of a specific nucleotide [35]. For instance, binding of dGTP at the allosteric site activates the enzyme for the conversion of ATP to dATP [35]. Although RNRs have been studied extensively for their biological role and reaction mechanism, no applications in biocatalysis were reported so far.

**Figure 1:**
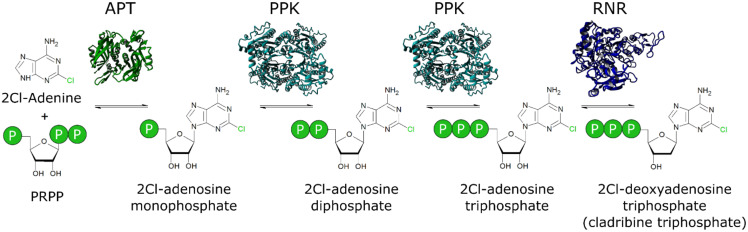
A novel cascade for the biosynthesis of non-natural deoxyribonucleotides: The adenine phosphoribosyltransferase (APT) catalyzes the loading of the nucleobase 2Cl-adenine on the ribose moiety in the form of phosphoribosyl pyrophosphate (PRPP). The polyphosphate kinase (PPK) performs two consecutive phosphorylation reactions to form 2Cl-adenosine triphosphate. The ribonucleotide reductase (RNR) catalyze the reduction to the final product 2Cl-deoxyadenosine-triphosphate.

In this article, we report a new biocatalytic approach for the biosynthesis of non-natural deoxyribonucleotides. The three enzymes adenosine phosphoribosyltransferase (APT), polyphosphate kinase (PPK), and ribonucleotide reductase (RNR) were combined to form a one-pot enzyme cascade for the production of cladribine-triphosphate from the generic educts PRPP and 2Cl-adenine (Figure 1). The objective of this study is to explore the feasibility of this cascade for the small-scale production of cladribine triphosphate, the active metabolite of the anti-cancer, and multiple sclerosis drug cladribine.

## 2. Materials and Methods

### 2.1. General Information

If not stated differently, all used chemicals were purchased from Sigma-Aldrich (Steinheim, Germany) or Carl Roth (Karlsruhe, Germany). Nucleotides and deoxyribonucleotides were purchased from Jena Bioscience (Jena, Germany).

### 2.2. Enzyme Expression and Purification

Three different enzymes were produced by recombinant expression in *Escherichia coli*. Details about the enzymes with origins, accession numbers, and references are given in Table 1. All three genes reside in a pET28b(+) expression vector with an N-terminal His-tag. Expression was performed in *E. coli* BL21(DE3) by growing a 600 mL culture with LB medium (30 µg ml^−1^ kanamycin) to an OD_600_ of 1.0 at 37 °C and 130 RPM. After induction with IPTG to a final concentration of 0.1 mmol L^−1^, expression was performed at 37 °C for 16 h (APT), at 20 °C for 22 h (PPK), and at 37 °C for 4 h (RNR). Cells were harvested by centrifugation and resuspended in His-wash buffer (50 mmol L^−1^ tris, 300 mmol L^−1^ NaCl, 10 mmol L^−1^ imidazole, pH = 8.0). Cell lysis was performed by high-pressure treatment (French press) three times at 10 MPa (Sim Aminco, Urbana, IL, USA). The lysate was centrifuged and applied on a HisTrap^TM^ FF crude 5 mL column (GE Healthcare, Chicago, IL, USA). After washing, elution was performed in His-elution buffer (50 mmol L^−1^ tris, 300 mmol L^−1^ NaCl, 500 mmol L^−1^ imidazole, pH = 8.0). The eluate was desalted via a HiPrep^TM^ 26/10 desalting column (GE Healthcare, Chicago, Illinois, USA) in desalting buffer (50 mmol L^−1^ tris, 300 mmol L^−1^ NaCl, pH = 8.0). To the desalted enzyme, glycerol was added to a final concentration of 20% (*v/v*). The samples were aliquoted, frozen in liquid nitrogen and stored at −80 °C. For the purification of the RNR, 1 mmol L^−1^ dithiothreitol (DTT) was added to all buffers.

### 2.3. Enzyme Activity Assays

Enzyme activity assays were performed under the same general conditions for all three enzymes. The standard reaction buffer consisted of 50 mmol L^−1^ tris and 20 mmol L^−1^ MgCl_2_ at a pH-value of 8.0. The reactions were performed at 40 °C for 3–15 min. The APT reactions were performed with 1 mmol L^−1^ adenine, 3 mmol L^−1^ phosphoribosyl pyrophosphate (PRPP), and 5 nmol L^−1^ enzyme. The PPK reactions with AMP as substrate were performed with 2 mmol L^−1^ AMP, 5 mmol L^−1^ sodium polyphosphate, and 10 nmol L^−1^ enzyme. For reactions with ADP, 2 mmol L^−1^ of the substrate and 100 nmol L^−1^ enzyme were applied. The RNR reactions were performed with 1 mmol L^−1^ ATP, 0.5 mmol L^−1^ dGTP, 10 mmol L^−1^ DTT, 4 µmol L^−1^ adenosylcobalamin, and 1 µmol L^−1^ enzyme. The reactions were started by addition of the respective enzyme. The reactions were quenched by adding 50 µL of the reaction to 50 µL methanol, vigorous mixing and incubation at 70 °C for 10 min, leading to immediate precipitation of the enzymes. After addition of 200 µL water and centrifugation, the concentration of the reaction product was determined via HPLC (2.5). Enzyme activity was calculated as the product formation over time.

### 2.4. Enzyme Cascade Reactions

The cascade reactions with adenine and 2Cl-adenine were performed under the following conditions. The reaction buffer consisted of 50 mmol L^−1^ tris and 20 mmol L^−1^ MgCl_2_, 3 mmol L^−1^ PRPP, 5 mmol L^−1^ sodium polyphosphate, 0.5 mmol L^−1^ dGTP, 10 mmol L^−1^ DTT, 6 µmol L^−1^ adenosylcobalamin at a pH-value of 8.0. The cascade reaction with adenine as initial substrate was performed with 1 mmol L^−1^ substrate, 10 nmol L^−1^ APT, 40 nmol L^−1^ PPK, and 2 µmol L^−1^ RNR. For the cascade reaction with 2Cl-adenine as initial substrate, 1 mmol L^−1^ 2Cl-adenine, 1 µmol L^−1^ APT, 400 nmol L^−1^ PPK, and 5 µmol L^−1^ RNR were applied. Both cascade reactions were started by addition of the enzymes and were incubated for 150 min at 40 °C. The reaction samples were quenched by adding 50 µL of the reaction to 50 µL methanol, vigorous mixing and incubation at 70 °C for 10 min, leading to immediate precipitation of the enzymes. After addition of 200 µL water and centrifugation, the concentration of the reaction product was determined via HPLC (2.5).

### 2.5. HPLC-Analytics

HPLC analysis was performed on a Knauer Azura^®^-HPLC (Knauer wissenschaftliche Geräte, Berlin, Germany) with a Eurosphere II 100-5 C18 column (Knauer wissenschaftliche Geräte, Berlin, Germany). The analytics for adenine and adenosine nucleotides were conducted at a flow rate of 0.5 mL min^−1^ with the following eluents: (A) 50 mmol L^−1^ KP_i_-Buffer (pH = 7.0), 10 mmol L^−1^ tetrabutylammonium hydroxide (TBAH), and 10% (*v/v*) methanol; (B) 50 mmol L^−1^ KP_i_-Buffer (pH = 7.0), 10 mmol L^−1^ TBAH, and 30% (*v/v*) methanol. The following elution profile was used: 0 min 60% A, 8 min 0% A, 18 min 0% A, 19 min 60% A, 22 min 60% A. The retention times of the measured components are 4.9 min (adenine), 9.0 min (AMP), 14.2 min (ADP), 18.0 min (ATP), and 19.7 min (dATP). Retention time definition and calibration was performed with analytical standards for each compound. 

The analytics for 2Cl-adenine and 2Cl-adenosine nucleotides were conducted at a flow rate of 0.4 mL min^−1^ with the following eluents: (A) 50 mmol L^−1^ KP_i_-Buffer (pH = 7.0), 10 mmol L^−1^ TBAH, and 10% (*v/v*) methanol; (B) 50 mmol L^−1^ KP_i_-Buffer (pH = 7.0), 10 mmol L^−1^ TBAH, and 35% (*v/v*) methanol. The following elution profile was used: 0 min 60% A, 24 min 0% A, 42 min 0% A, 43 min 60% A, 46 min 60% A. The retention times of the measured components are 12.0 min (2Cl-adenine), 21.2 min (2Cl-AMP), 25.2 min (2Cl-dAMP), 28.3 min (2Cl-ADP), 30.7 min (2Cl-dADP), 31.8 min (2Cl-ATP), 33.8 min (2Cl-dATP). Analytical standards were only available for 2Cl-adenine and 2Cl-deoxyadeonsine triphosphate. Other intermediates or products were identified by mass spectrometry on Thermo Fisher Q Exactive LC-MS (Thermo Fisher Scientific, Waltham, MA, USA). The calibrations for 2Cl-adenine and 2Cl-deoxyadenosine triphosphate were performed with analytical standards. Concentrations of the other compounds were estimated based on the calibration for 2Cl-deoxyadenosine triphosphate. This estimation should be valid considering the similar values (slopes) for the calibrations for adenosine nucleotides. The coefficient of variation lies at 6.9% with a tendency to higher values with increasing retention time. Details for the peak identification are given in the Appendix A (Figure A2 and Figure A3).

## 3. Results

**Figure 2:**
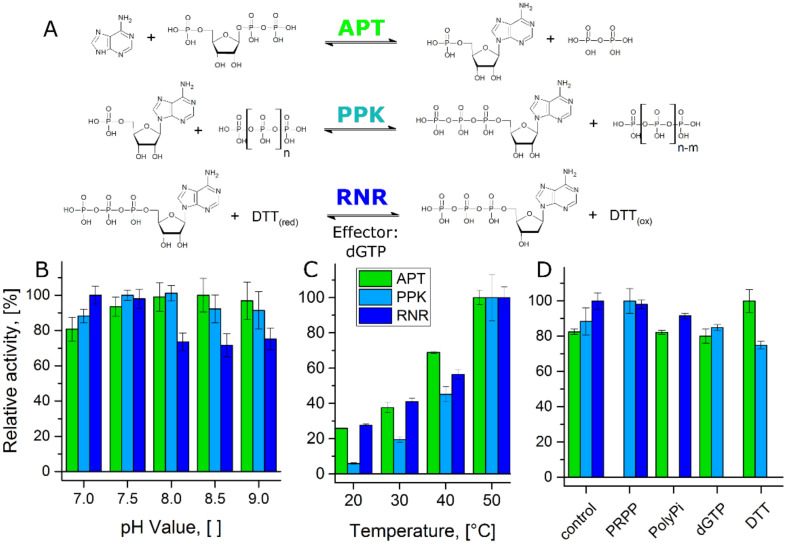
Compatibility test of the single reactions in the cascade: (**A**) Single reaction steps of the cascade with substrates, co-substrates, products, and effector. (**B**) Relative activity of the enzymes at 40 °C in a pH-range from 7.0 to 9.0. 100% relative activity corresponds to 324 U mg^−1^ (APT), 27 U mg^−1^ (PPK), and 0.52 U mg^−1^ (RNR), respectively. PPK activity was determined for the conversion of AMP to ADP. (**C**) Relative activity of the enzymes at a pH-value of 8.0 in a temperature range from 20 °C to 50 °C. 100% relative activity corresponds to 586 U mg^−1^ (APT), 55 U mg^−1^ (PPK), and 0.78 U mg^−1^ (RNR), respectively. PPK activity was determined for the conversion of AMP to ADP. (**D**) Relative activity of the enzymes at 40 °C and a pH-value of 8.0 in the presence of substrates and effector from the other reaction steps. 100% relative activity corresponds to 391 U mg^−1^ (APT), 31 U mg^−1^ (PPK), and 0.44 U mg^−1^ (RNR), respectively. PPK activity was determined for the conversion of AMP to ADP.

The establishment of a cascade reaction requires the enzymes to be compatible with each other in two fashions. First, the enzymes must function under similar reaction conditions. Second, the respective reactants of each single reaction step must not interfere with the other steps of the cascade (Figure 2A). To address the first point, the three enzymes APT, PPK, and RNR were tested for their respective activities at different pH-values and reaction temperatures in a tris-buffer system. All reactions were performed in the presence of MgCl_2_, since APT and PPK are Mg^2+^-dependent enzymes and nucleotide triphosphates are stabilized as well [29,37].

All enzymes showed stable activities between the pH-values 7.0 and 9.0 with optima at 8.5 (APT), 8.0 (PPK), and 7.0 (RNR), respectively (Figure 2B). Only the RNR activity was reduced at higher pH-values. The cascade was designed for a conversion of 2Cl-adenine, which is only poorly soluble in water at a neutral pH-value. Considering enzyme activity and substrate solubility, we selected a pH-value of 8.0 for the cascade reaction.

**Figure A1:**
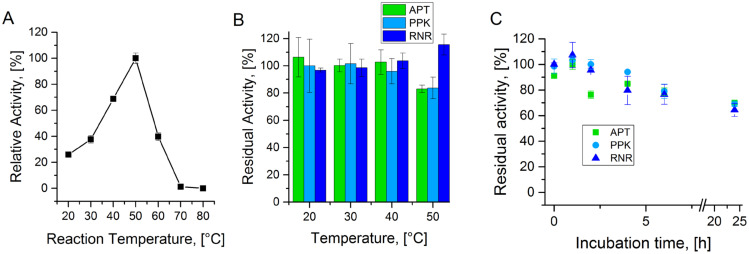
Enzyme temperature stability: (**A**) Relative activity of APT at a pH-value of 8.0 in a temperature range from 20 °C to 80 °C. (**B**) Residual activity of APT, PPK, and RNR after 1 h of incubation at the respective temperatures. (**C**) Residual activity of APT, PPK, and RNR after incubation at 40 °C, measured at several time points up to 24 h.

**Figure A2:**
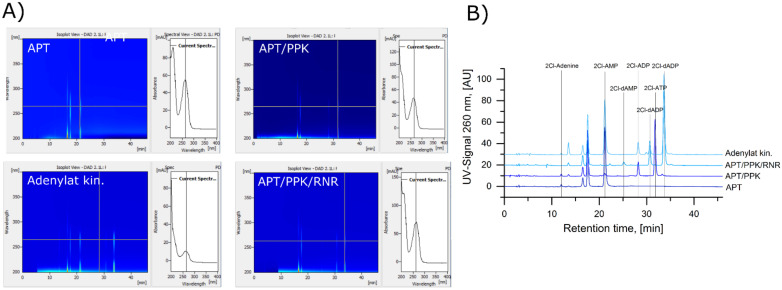
HPLC Separation of 2Cl-adenine and 2Cl-adenosine nucleotides: (**A**) UV spectra of the chromatographic separations of the reactions with APT alone (APT), APT and PPK (APT/PPK), the adenylate kinase reaction (Adenylate kin.), and the full cascade with all three enzymes (APT/PPK/RNR). 2Cl-adenine and the 2Cl-adenosine (deoxy)ribonucleotides show a specific absorption maximum at 264 nm. (**B**) Chromatograms of the reactions with APT alone (APT), APT and PPK (APT/PPK), the adenylate kinase reaction (Adenylate kin.), and the full cascade with all three enzymes at a wavelength of 260 nm. The peaks at 17 and 18 min are the reducing agent DTT and the effector dGTP, respectively.

**Figure A3:**
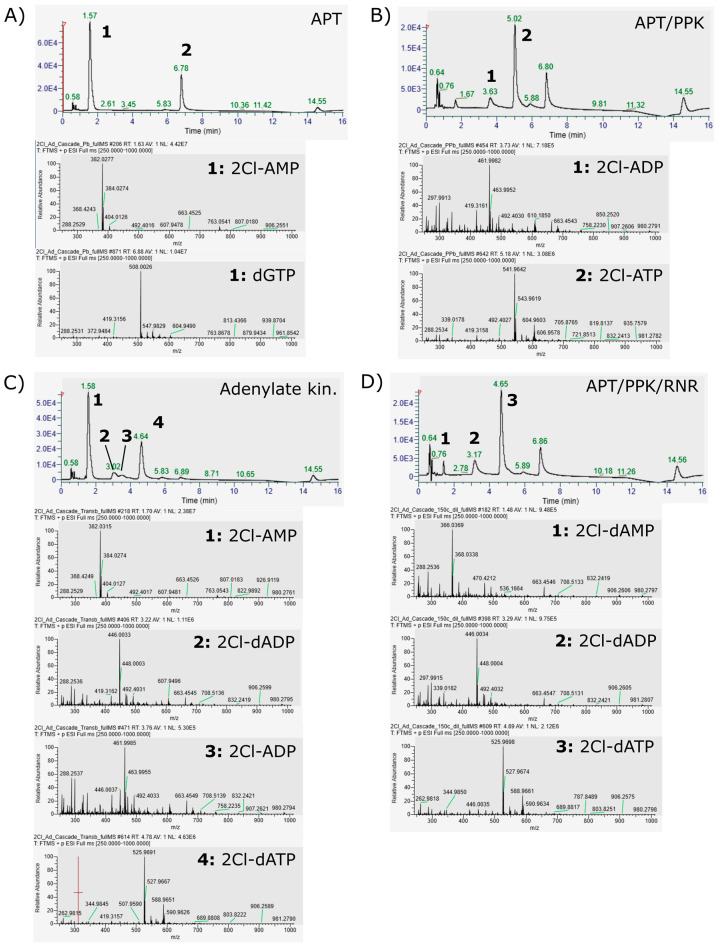
LC-MS identification of 2Cl-adenosine (deoxy)ribonucleotides: (**A**) LC and full-MS spectra of the reaction of APT only with 2Cl-adenine as substrate. Identified peaks: 1: 1.57 min, 2Cl-AMP; 2: 5.78 min, dGTP. (**B**) LC and full-MS spectra of the reaction of APT and PPK with 2Cl-adenine as substrate. Identified peaks: 1: 3.63 min, 2Cl-ADP; 2: 5.02 min, 2Cl-ATP. (**C**) LC and full-MS spectra of the adenylate kinase test reaction of APT and PPK with 2Cl-adenine as substrate and 2Cl-dATP as phosphate donor. Identified peaks: 1: 1.58 min, 2Cl-AMP; 2: 3.02 min, 2Cl-dADP. 3.51 min, 2Cl-ADP; 4.64 min. 2CL-dATP. (**D**) LC and full-MS spectra of the Reaction of the full cascade with 2Cl-adenine as substrate. Identified peaks as 1: 1.48 min, 2Cl-dAMP; 2: 3.17 min, 2Cl-dADP; 3: 4.65 min, 2Cl-dATP.

The published optimal reaction temperatures of PPK and RNR are 70 °C for both enzymes [29,32]. For APT, the optimal reaction temperature was determined to be 50 °C in a preliminary experiment (Figure A1A). The temperature dependent activities of the three enzymes at the reaction conditions of the cascade were tested between 20 and 50 °C (Figure 2C). All three enzymes showed an increase in activity over this temperature range. Since particularly APT and PPK showed higher stability at 40 °C (Figure A1B), this temperature was selected as reaction temperature for the cascade.

The question whether the reactants of the single reactions interfere with other reaction steps was addressed by testing each individual enzyme in the presence of the co-substrates of the other reactions and the RNR-effector dGTP (Figure 2D). None of the tested compounds had a major inhibitory effect on the other enzymes. The most pronounced effect was actually an increase of the APT activity in the presence of DTT, which functions as reducing agent in the RNR reaction.

After establishing the reaction conditions for the cascade, the kinetic parameters of the enzymes were determined for each reaction step under the selected reaction conditions (Table 2). Since the PPK is meant to catalyze two consecutive phosphorylation reactions, the kinetic parameters for both, the phosphorylation of AMP and ADP were determined separately. APT and PPK showed high k_cat_- and K_M_-values in the 10 µmol L^−1^ range for the first two reaction steps. The PPK catalyzed ADP phosphorylation and the RNR reaction showed K_M_-values in the 100 µmol L^−1^ range with k_cat_ values of 0.43 ± 0.02 s^−1^ and 0.56 ± 0.02 s^−1^, respectively. These differences in catalytic performance and the position in the cascade were considered for the selection of the enzyme concentrations in the cascade.

**Figure 3:**
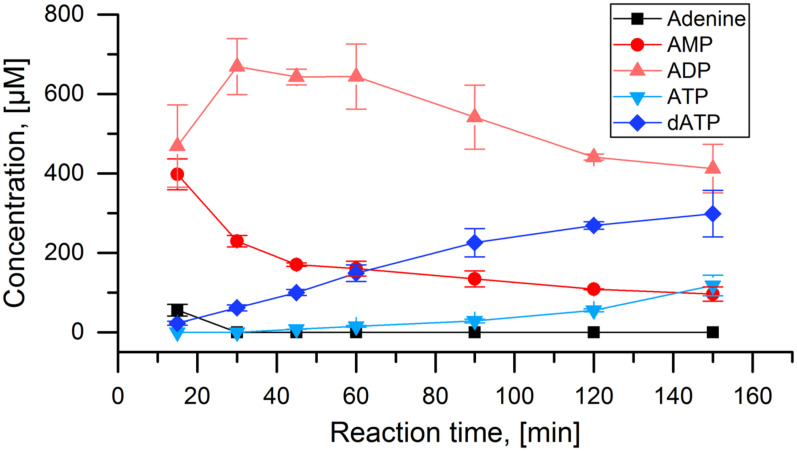
Test of the APT-PPK-RNR reaction cascade with adenine: Concentrations of adenine and all detected adenosine nucleotides over the time course of the full cascade reaction with 1 mmol L^−1^ adenine as initial substrate. Applied enzyme concentration: APT: 10 nmol L^−1^, PPK: 40 nmol L^−1^, RNR: 2 µmol L^−1^.

The general functionality of the cascade was tested with adenosine as initial substrate, where each enzyme is known to perform their respective part of the reaction. The cascade reaction showed near complete depletion of adenine after 15 min with equal amounts of AMP and ADP being produced (Figure 3). Over the next two hours, a steady increase in the concentration of dATP was observed. A minor accumulation of ATP is visible in the last 30 min of the experiment. Overall, a dATP concentration of 300 µmol L^−1^ was achieved, corresponding to a final reaction yield of 30% after 150 min.

**Figure 4:**
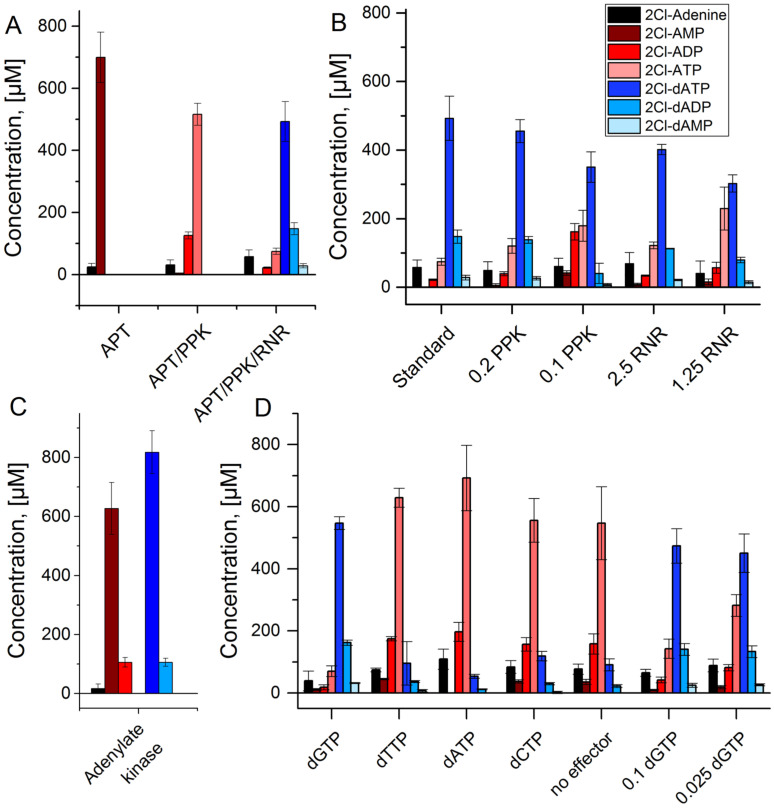
APT-PPK-RNR reaction cascade with 2Cl-adenine as substrate: (**A**) Stepwise assembly of the reaction cascade with APT alone (APT), APT and PPK (APT/PPK), and the full cascade of APT, PPK, and RNR. (**B**) Full reaction cascade with reduced PPK and RNR concentrations in µmol L^−1^; standard concentrations are 1 µmol L^−1^ (APT), 0.4 µmol L^−1^ (PPK), and 5 µmol L^−1^ (RNR), respectively. (**C**) Adenylate kinase reaction test with APT (1 µmol L^−1^) and PPK (0.4 µmol L^−1^) starting with 1 mmol L^−1^ 2Cl-adenine and 2Cl-deoxyadenosine triphosphate each. (**D**) Full reaction cascade with different effector molecules (0.5 mmol L^−1^) and different concentrations of the effector dGTP in mmol L^−1^.

Applying the same conditions for the cascade with 2Cl-adenine did not yield any detectable conversion of the initial substrate. Thus, enzyme concentrations were adjusted for each reaction step separately, to account for reduced activities of the enzymes for the conversion of the non-natural substrate. It is necessary to point out, that the concentrations of all 2Cl-adenosine nucleotides, except for 2Cl-dATP, are estimations based on the HPLC calibration for 2Cl-dATP (for details see Section 2). For the APT reaction, increasing the enzyme concentration to 1 µmol L^−1^ led to near complete depletion of 2Cl-adenine after 60 min (Figure 4A). Application of 1 µmol L^−1^ APT and 0.4 µmol L^−1^ PPK led to high conversions to 2Cl-ATP in the same reaction time. The same concentrations of APT and PPK with addition of 5 µmol L^−1^ RNR led to the production of 500 µmol L^−1^ of the desired product 2Cl-dATP within 60 min (Figure 4A). Reducing the enzyme concentrations of RNR or PPK led to decreasing production of 2Cl-dATP (Figure 4B). In addition to the desired reaction product, the formation of 2Cl-dADP and 2Cl-dAMP was observed.

One cause for the occurrence of these side products may be the hydrolysis of the 2Cl-dATP but another possibility is an adenylate kinase reaction catalyzed by PPK. To test this hypothesis, a reaction with APT and PPK was performed, replacing polyphosphate by 2Cl-dATP as phosphate donor (Figure 4C). After 60 min reaction time, similar amounts of 2Cl-dADP and 2Cl-ADP were observed, indicating the transfer of a phosphate group from 2Cl-dATP to 2Cl-AMP.

The RNR reaction requires an effector molecule for the conversion of a specific substrate. Initially, the effector dGTP was applied because it activates the enzyme for the conversion of ATP. Since dGTP is not necessarily the best effector for the non-natural substrate 2Cl-ATP, the other natural effectors dATP, dTTP, and dCTP were tested. All effectors except for dGTP led to a strongly decreased 2Cl-dATP synthesis in combination with an accumulation of 2Cl-ATP, comparable to the control without effector (Figure 4D). A decrease of the concentration of dGTP was possible with moderate reduction of 2Cl-dATP synthesis.

**Figure 5:**
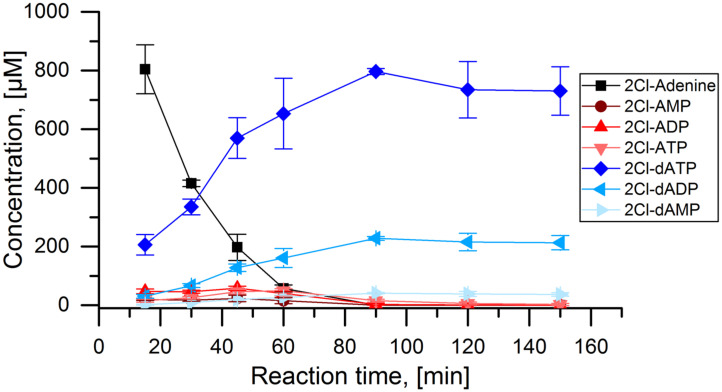
APT-PPK-RNR reaction cascade with 2Cl-adenine as substrate: Concentrations of 2Cl-adenine and all detected 2Cl-adenosine nucleotides over the time course of the full cascade reaction with 1 mmol L^−1^ 2Cl-adenine as initial substrate. Applied enzyme concentration: APT: 1 µmol L^−1^, PPK: 400 nmol L^−1^, RNR: 5 µmol L^−1^.

With the conditions for the cascade established above, it was tested over a reaction time of 150 min (Figure 5). The initial substrate 2Cl-adenine was depleted after 60 min. The concentrations of 2Cl-AMP, 2Cl-ADP, and 2Cl-ATP were constantly low, peaking around 60 min. The desired product 2Cl-dATP was produced constantly up until 90 min with a final concentration of 800 µmol L^−1^. In parallel, also 2Cl-dADP and to a small extent 2Cl-dAMP were produced. After 90 min, the concentrations of all components remained stable. A final reaction yield of 80% was achieved for the main product 2Cl-dATP or cladribine triphosphate with 2Cl-dADP being one major side product.

## 4. Discussion

This study combines three well studied, but so far unrelated, nucleotide-modifying enzymes to create a novel one-pot enzyme cascade for the biosynthesis of cladribine triphosphate. Phosphoribosyltransferases are a well-established tool for nucleoside monophosphate biosynthesis and have been applied in numerous studies [12,13,14,15,16,17]. Polyphosphate kinases (PPK) have been studied primarily with respect to cofactor regeneration [22,23,24,25,26,27]. Recently, they were also investigated for their capability to produce adenosine tetra- and pentaphosphate [28]. In our work, we use a PPK in new fashion, to catalyze the phosphorylation of a non-natural nucleotide. Ribonucleotide reductases (RNR) are a well-studied class of enzymes in terms of their catalytic mechanism and biological function [34]. They have been used as model enzymes to investigate radical-based catalysis in enzymes as well as evolutionary aspects [31]. However, no biocatalytical applications of this class of enzymes have been reported up to this study.

The functionality of the novel cascade was tested with the natural substrate adenine and the non-natural substrate 2Cl-adenine. While all three enzymes were capable of the conversion of 2Cl-adenine or 2Cl-adenosine compounds, APT showed strongly reduced activity for 2Cl-adenine. A 100-fold concentration of the enzyme was required compared to the natural substrate to reach similar conversions. PPK and RNR were used in higher concentrations as well (10× and 2.5×, respectively), but also higher conversions were achieved for the respective 2Cl-adenosine substrates. This indicates higher tolerance of the latter two enzymes of the cascade for these non-natural substrates. Purification of the reaction intermediates to study the single reaction steps will allow a more detailed analysis of the respective substrate promiscuity.

The cascade established in this study, is the first enzyme cascade, proven capable of the production of 2Cl-deoxyadenosine triphosphate or cladribine triphosphate, extending the accessibility of this important class of compounds. The overall reaction yield of the cascade was 800 µmol L^−1^ of the desired main product after 90 min reaction time. With 2Cl-deoxyadenosine diphosphate, one major side product was formed with an estimated concentration of 200 µmol L^−1^. Since cladribine triphosphate has not been produced in a biocatalytical cascade before, we assessed the performance of this cascade by comparing the yield and purity with other biocatalytical cascades that produce NTPs or dNTPs.

5F-UTP and 8-Azaguanine were produced by biocatalytical cascades with phosphoribosyltransferases followed by phosphorylation with different kinases. In both cascades, PRPP was generated in situ by a ribokinase and a PRPP synthetase. For 5F-UTP, an isolated yield of 80% corresponding to a product concentration of 800 µmol L^−1^ was achieved after 110 h [19]. For 8-Azaguanine, an isolated yield of 60% corresponding to a product concentration of 1200 µmol L^−1^ was achieved after 240 h [18]. Both cascades use 0.01–1.5 U mL^−1^ of the different enzymes compared to 6.5 (APT), 0.4 (PPK), and 0.2 (RNR) U mL^−1^ in this study. Nucleotide concentrations in the reactions are comparable but the isolated yields cannot be directly compared to the reaction yield in our study. In a more recent article, different kinases have been applied for the synthesis of the natural deoxyribonucleotides dATP and dCTP from the corresponding nucleosides [21]. A total of 1 mmol L^−1^ of the respective deoxyribonucleoside was converted by three kinases with concentrations between 0.016 and 0.02 mg ml^−1^, compared to 0.02 (APT), 0.015 (PPK), and 0.37 (RNR) mg mL^−1^ in this study. Reaction yields of 97% and 60% were obtained after 19 h for dCTP and dATP, respectively. In conclusion, the cascade presented in this study enables the biosynthesis of cladribine triphosphate, achieving comparable product concentrations and yields of established systems for other nucleotides. In its current setup, more enzyme is applied in average than in other studies but the maximal yield is already reached after 90 min compared to 19–240 h.

The novel cascade has three main points to address to further improve its performance and feasibility for the biosynthesis of non-natural deoxyribonucleotides:

(1) PRPP as substrate is an expensive co-substrate. Although the value of the synthesized cladribine triphosphate may justify that, an *in situ* generation of PRPP would render the process more feasible. Ribokinases and PRPP synthetases may be used for this end as described in other studies, given that these enzymes are compatible with the current cascade [15,18,20,38].
(2) The adenylate kinase reaction of the PPK that was used in this cascade has been demonstrated for the reaction ATP + AMP → 2 ADP before [28]. This reaction seems to be the main reason for the formation of side product 2Cl-deoxyadenosine diphosphate (Figure 4C and Figure 5). Since it is a competing reaction to the polyphosphate dependent phosphorylation, it may be possible to reduce this side reaction by optimization of PPK and polyphosphate concentrations. Enzyme engineering of the PPK would be a possible way to reduce this side reaction as well. Formation of 2Cl-dADP by RNR catalyzed 2CL-ADP reduction can be excluded as a source for dADP generation, since the applied enzyme is a strict nucleoside triphosphate reducing RNR [32].
(3) The RNR requires an allosteric effector molecule to reach high activities for the reduction of a specific nucleotide. The effector, being a dNTP itself, is a rather expensive additive to the reaction cascade. Reduction of the concentration is possible for the price of a reduced RNR activity. One possibility to circumvent this issue would be the immobilization of the effector on the enzyme, as shown for NADH on different dehydrogenases [39]. By re-engineering of the effector binding site, the dependence of the enzyme on the allosteric effector might be deleted.

The design of the cascade also offers some inherent advantages over other cascade designs:

(1) In order to set up the cascade for 2Cl-adenine, it was assembled step by step to establish the required enzyme concentrations. However, each of these reactions could be a valuable biocatalytic process in its own right. Optimizing the reaction shown in Figure 4A could deliver a feasible process for the synthesis of 2Cl-adenosine triphosphate. Using the PPK2-II from *Acetinobacter johnsonii*, which is slower in the phosphorylation of the nucleoside diphosphate, could be used to synthesize 2Cl-adenosine diphosphate [28,40].
(2) The cascade may not be limited to the synthesis of cladribine triphosphate. Although the APT seems to be quite specific for adenosine, there are different phosphoribosyltransferases that cover different ranges of nucleobases. Examples are uracil phosphoribosyltransferases (UPT) or hypoxanthine(-guanine) phosphoribosyltransferases (H(G)PT) [17,38,41,42]. PPKs and RNRs are capable of the conversion of different natural nucleotides, with both purine and pyrimidine bases [29,32,35]. Therefore, a certain degree of substrate promiscuity towards non-natural nucleotides can be expected from both enzymes.

In this work, we demonstrated the application of a novel one-pot enzyme cascade for the challenging biosynthesis of cladribine triphosphate, the active compound of the anti-cancer and multiple sclerosis drug cladribine. While some aspects of the cascade may be improved, its performance is comparable to existing enzyme cascades for other nucleotides. Future experiments will show to what extent this cascade or parts of it may be useful for the biosynthesis of other important deoxyribonucleotides.

## Figures and Tables

**Table 1:** Nucleotide-modifying enzymes used in this study.

Enzyme	Name	Source	Uniprot ID	Reference
APT	*EcAPT*	*Escherichia coli*	P69503	[36]
PPK	*MrPPK*	*Meiothermus ruber*	M9XB82	[28,29,30]
RNR	TVNrdJm	Thermus virus TV74-23	A7XXH5	[32]

**Table 2:** **Kinetic parameters of the enzymes**: The kinetic parameters K_M_ and k_cat_ were determined for each enzyme and reaction step. Since the PPK catalyzes two reaction steps, the kinetic parameters were determined for each separately.

Enzyme	Substrate	K_M_, [µmol L^−1^]	k_cat_, [s^−1^]
APT	adenine	11.8 ± 5.2	119 ± 14
PPK	AMP	41 ± 6.3	17 ± 1.3
PPK	ADP	144 ± 14	0.43 ± 0.02
RNR	ATP	199 ± 13	0.56 ± 0.02

## Data Availability

All relevant data are contained in the manuscript and the Appendix A.

## Appendix A

### A.1. Temperature Stability of Enzymes from the Cascade

The three enzymes used in the cascade show temperature optima at 70 °C for the RNR and the MrPPK [29,32] and 50 °C for the APT (Figure A1A). For all three enzymes, the temperature stabilities were determined. The enzymes were incubated for 1 h at the respective temperature and residual activities were determined (Figure A1B). The RNR shows no inactivation up until 50 °C. However, both APT and PPK show stable activities until 40 °C with a loss of 15% at 50 °C. The long-term stability of the enzymes at 40 °C was tested by incubating the enzymes at 40 °C for 24 h and measurement of the residual activity at different time points (Figure A1C). All three enzyme showed a slow inactivation at 40 °C with residual activities between 64% and 69% after 24 h.

### A.2. Identification of 2Cl-Adenosine Reaction Intermediates and Products

The cascade described in this article involves several 2Cl-adenonsine derivatives as intermediates and products. Since analytical standards are only available for 2Cl-adenine and 2Cl-deoxyadenosine triphosphate, all other compounds had to be identified by LC-MS from different reactions performed in this study.

Four different reactions were selected to identify all occurring compounds. Reactions with only part of the enzymes of the cascade (Figure 4A) were used to identify the intermediate products. The adenylate kinase test reaction (Figure 4C) and the samples of the full cascade after 150 min (Figure 5) were used to identify the 2Cl-deoxyadenosine side products. The UV spectra of the reactions from the HPLC method, described in 2.5, show multiple peaks with a common absorption maximum at 264 nm, corresponding to the absorption maximum of the 2Cl-adenine moiety (Figure A2), indicating the presence of a Cl-adenine derivative.

The same reactions were analyzed by LC-MS to identify the respective compounds. Since the HPLC method used so far is not compatible with mass spectrometry, a cHILIC column was used for separation of the nucleotide compounds. The method is describe in the last section of this appendix. Figure A3 shows the HPLC chromatograms recorded for the four reactions described above, in addition to the full mass spectra of the interesting chromatographic peaks (Figure A3). The reaction with only APT shows two main chromatographic peaks. The mass spectrum at 1.57 min retention time shows two main mass peaks at 382 and 384, which is in accordance with 2Cl-AMP with the chlorine isotopes ^35^Cl and ^37^Cl (Figure A3A). Accordingly, 2Cl-ADP and 2Cl-ATP were identified in the reaction with APT and PPK (Figure A3B). In the adenylate kinase reaction, the intermediate 2Cl-dADP was identified with mass peaks at 446 and 448 (Figure 4C). In the full cascade reaction the side products 2Cl-dADP and 2Cl-dAMP were identified next to the main product 2Cl-dATP.

### A.3. HPLC MS Method

HPLC separation was performed on a Thermo vanquish-HPLC (Thermo Fisher Scientific, Waltham, MA, USA) with a ZIC^®^-cHILIC 3 µm, 100 Å column (Merck Millipore, Burlington, MA, USA). The samples were prepared by diluting the HPLC samples, prepared for the abovementioned HPLC method (2.5), with two volumes acetonitrile. Separation was conducted at a flow rate of 0.3 mL min^−1^ with the following eluents: (A) 10 mmol L^−1^ ammonium acetate buffer (pH = 6.8) and 75% (*v/v*) acetonitrile; (B) 10 mmol L^−1^ ammonium acetate buffer (pH = 6.8) and 0% (*v/v*) acetonitrile. The following elution profile was used: 0 min 100% A, 10 min 70% A, 12 min 25% A, 13 min 25% A, 13.5 min 100% A, 16 min 100% A.

Mass spectrometric analysis was performed on a coupled Thermo Q Exactive Mass spectrometer (Thermo Fisher Scientific, Waltham, MA, USA). The analysis was performed with the following scan parameters: Polarity: Positive, AGC target: 3 × 10^6^, maximum IT: 200 ms; scan range: 250 to 1000 m/z. Resolution 70,000. The electron spray settings were spray voltage: 4 kV; capillary temperature 320 °C; sheath gas flow rate: 25 mL min^−1^; AUX gas flow rate: 10 mL min^−1^; S-lens RF level: 55.
